# The Diphtheria Bacillus and Its Media

**Published:** 1912-05-25

**Authors:** T. Thomson Rankin

**Affiliations:** Senior A.M.O., City Hospitals, Leeds.


### DIPHTHERITIC CONVALESCENTS.

### The Diphtheria Bacillus and its Media.

By T. THOMSON RANKIN, M.D., Senior A.M.O., City Hospitals, Leeds.

The examination of patients convalescent from diphtheria is of great importance, and a bacteriological examination is the only reliable means by which it is possible to assume that a patient is not a carrier. Any method that will simplify this procedure without the sacrifice of efficiency is to be seriously considered and thoroughly investigated. Carriers of diseases are undoubtedly the source of many cases that otherwise would not occur, and it is the duty of the physician to reduce or eradicate them. This requires careful bacteriological examination, and the provision of means to isolate carriers until they are free from specific germs. Diphtheria carriers are by no means uncommon, and it is essential that no case should be discharged from hospital that could act as a real or potential carrier. The term potential carrier refers to cases that show on examination a preponderance of Hofmann bacilli. Such patients are undoubtedly liable to diphtheria, and should be regarded as potential carriers.

### A Lesson from Leeds.

In this hospital it has been noticed that many patients having a superabundance of Hofmann bacilli in their throat, and showing no diphtheria bacilli after careful and repeated examinations, are liable to diphtheria. The cause of this is not known, and the proof is more clinical than bacteriological. Most evidence is against any direct relationship between the bacillus of Hofmann and *B. diphtheriæ*, but there is undoubtedly some relation. It may be said that the *B. diphtheriæ* were few and had been overlooked. This might possibly occur in a few instances, but not in all the cases observed here. The chief interest, however, lies in the fact that such cases occur, and due attention should be paid to them. To determine when a patient is free from such danger necessitates repeated bacteriological examinations of the throat or other part that was originally affected. It is advisable, however, to examine the throat in all cases, no matter where the original lesion was situated.

One or two negative bacteriological examinations are not sufficient; three at least are essential. The sources of error are numerous, and it is only when one obtains three successive negative results that one can assume, with any degree of certainty, that the patient is not a carrier. The terms positive and negative refer respectively to the presence or absence of the organism of diphtheria. One negative result is of practically no use. It is very common to get a premature negative culture. This may be due to any of the following causes:—

1. Less care may be taken than was the case with the first swabbing of the throat.
2. The diphtheria organisms may have disappeared temporarily from the part swabbed.
3. The bacillus may not be removed from the part, or may not be transferred to the medium.
4. The organism may fail to grow on the medium, or may be overgrown and overlooked.

A negative culture between two positive ones is by no means rare. It is thus seen that the value of one negative examination for discharge purposes is practically nil.

Two successive negative cultures are of more value than one, but even two are barely sufficient. The points that militate against one negative result also apply in this case, but to a lesser degree. One also finds that two successive negative cultures are sometimes followed by one showing the *Bacillus diphtheriæ*.

Three successive negative cultures are recommended as the lowest limit compatible with safety. It may be necessary in some cases to increase this number. Rarely, if ever, does one find three negative cultures taken successively at intervals of three days followed by a positive one, provided proper care is exercised in the swabbing of the throat and in the examination of the growth.

Great care must be exercised when one is making an examination of the eye, ear, nose, and skin for the Klebs-Loeffler bacillus, as organisms presenting analogous morphological and cultural characters are found in these situations. Only by the reaction of these organisms on the various sugar media and their action on guinea-pigs can one distinguish them from *B. diphtheriæ*.

The throat is the most common situation for diphtheria, and all subsequent remarks will apply to this part.

Before it is possible to grow the Klebs-Loeffler bacillus, it is necessary to have a suitable medium. The ordinary media—agar, glycerin agar, and blood serum—are very good and easily made. *B. diphtheriæ* grows well on them, as do the other micro-organisms found in the throat. Other media have been introduced at various times with the view of aiding the growth of the diphtheria bacillus, and of these the best are Loeffler's and Lorrain Smith's. For routine purposes the former is the better, and is not difficult to prepare.

### A Useful Medium Described.

None of the media mentioned above have any very great selective properties. Selective media have not been much in evidence as far as the diphtheria bacillus is concerned. The following medium has been in constant use in this hospital for nearly two years, and has been found very satisfactory. It has certain selective properties and, in other respects, is as good as any of the above-mentioned media. It consists of:—

| | |
|---|---|
| Blood serum of the sheep ... ... ... | 3 parts |
| Broth, sterile water, or a mixture of broth and water ... ... ... ... ... | 1 part |
| Glucose ... ... ... ... ... ... | 0.5 per cent. |
| Potassium sulphocyanide ... ... ... | 1 per cent. |
| 0.5 per cent. solution (watery) of neutral red | 2 per cent. |

The original medium contained broth, but, subsequently, it was found that sterile water was quite as good. A mixture of broth and water of any strength can also be used. This medium is easy to make and enables anyone to say at a glance if the diphtheria bacillus is absent. When there is none present, there is no red colouration of the medium.

The colouring of the medium depends on the production of acid or alkali by the organisms present. Acid-producing organisms will produce a red colour, and those producing an alkali a yellow colour. It is well known that *B. diphtheriæ* produces acid in the presence of glucose, and this property is made use of. The great value of the medium is its negative feature, allowing one to say when the Klebs-Loeffler bacillus is absent, but it has also much value positively, as one can tell, after some practice, when this organism is present. This is done by noting the intensity, extent, and tint of the red colour produced. Experience soon enables one to distinguish the colours due to various cocci and that due to the diphtheria bacillus. When this power has been acquired, one can dispense with the microscope and save much time, especially if one has a large number of cultures to examine. The number of tubes of this medium examined in this hospital is well over ten thousand, and the results obtained have justified its continued use.

